# Advisory opinion of the AWMF Ad hoc Commission In-vitro Diagnostic Medical Devices regarding in-vitro diagnostic medical devices manufactured and used only within health institutions established in the Union according to Regulation (EU) 2017/746 (IVDR)

**DOI:** 10.3205/000295

**Published:** 2021-06-01

**Authors:** Petra Hoffmüller, Monika Brüggemann, Thomas Eggermann, Kamran Ghoreschi, Detlef Haase, Jörg Hofmann, Klaus-Peter Hunfeld, Katharina Koch, Christian Meisel, Patrick Michl, Jens Müller, Carsten Müller, Holger F. Rabenau, Dirk Reinhardt, Markus J. Riemenschneider, Ulrich J. Sachs, Ulrich Sack, Albrecht Stenzinger, Thomas Streichert, Nils von Neuhoff, Wilko Weichert, Christof Weinstock, Stefan Zimmermann, Folker Spitzenberger, AWMF Ad hoc Commision In-vitro Diagnostic Medical Devices

**Affiliations:** 1 Deutsche Gesellschaft für Humangenetik (GfH); 2 Deutsche Gesellschaft für Hämatologie und Medizinische Onkologie (DGHO); 3 Deutsche Gesellschaft für Dermatologie (DDG); 4 Gesellschaft für Virologie (GfV); 5 Deutsche Gesellschaft für Hygiene und Mikrobiologie (DGHM); 6 Gesellschaft zur Förderung der Qualitätssicherung in medizinischen Laboratorien (INSTAND e.V.); 7 Gesellschaft für Toxikologische und Forensische Chemie (GTFCh); 8 Deutsche Gesellschaft für Immunologie (DGfI); 9 Deutsche Gesellschaft für Gastroenterologie, Verdauung und Stoffwechsel (DGVS); 10 Gesellschaft für Thrombose- und Hämostaseforschung (GTH); 11 Deutsche Gesellschaft für Klinische Pharmakologie und Therapie (DGKliPa); 12 Deutsche Gesellschaft für Klinische Chemie und Laboratoriumsmedizin (DGKL); 13 Gesellschaft für pädiatrische Onkologie und Hämatologie (GPOH); 14 Deutsche Gesellschaft für Neuropathologie und Neuroanatomie (DGNN); 15 Deutsche Gesellschaft für Pathologie (DGP); 16 Deutsche Gesellschaft für Transfusionsmedizin und Immunhämatologie (DGTI); 17 Paul-Ehrlich-Gesellschaft (PEG); 18 Deutsche Gesellschaft für Pharmazeutische Medizin (DGPharMed)

**Keywords:** IVDR, laboratory-developed tests, in-house IVD, validation, performance evaluation, quality management

## Abstract

In view of the approaching application date of Regulation (EU) 2017/746 („IVDR“) and the resulting EU-wide, harmonized requirements for in-vitro diagnostic medical devices (IVD) manufactured and used within European health institutions, the Ad hoc Commission IVD of the German Association of the Scientific Medical Societies (AWMF) takes a national position on the details of the requirements and conditions related to the use of these IVD products.

The Ad hoc Commission IVD emphasizes the relevance of examination procedures developed in medical laboratories, especially in the field of orphan diseases and new diagnostic markers. The IVDR sets an adequate regulatory framework for IVD manufactured and used within health institutions as long as these requirements are fulfilled with reliability and in accordance with the current state of the art in medical laboratory sciences. At the same time, the IVDR requirements have to be regarded under a pragmatic view and in accordance with the quality management systems approved within the different EU Member States. On the one hand, the mandatory requirements of the RiLiBÄK play an essential role in Germany. On the other hand, elements of voluntarily applicable international standards may support the fulfilment of product requirements for safety and performance according to Annex I of the IVDR. Both the complexity and possible solutions for the implementation of the IVDR requirements are discussed on the basis of examples such as the required documentation, performance evaluation and software validation.

The Ad hoc Commission IVD recommends that, while aiming at a preferably EU-wide harmonized interpretation of the IVDR requirements, the flexibility in medical laboratory diagnostics necessary for patient care, including the use of IVD from in-house production, should be emphasized.

## Zusammenfassung

Vor dem Hintergrund des nahenden Geltungsbeginns der Verordnung (EU) 2017/746 („IVDR“) und der damit EU-weit harmonisierten Anforderungen an In-vitro-Diagnostika (IVD) aus Eigenherstellung positioniert sich die Ad-hoc-Kommission In-vitro-Diagnostika der Arbeitsgemeinschaft der Wissenschaftlichen Medizinischen Fachgesellschaften (AWMF) im Einzelnen zu den in der IVDR gestellten Anforderungen und Bedingungen zur Verwendung dieser Produkte.

Die Ad-hoc-Kommission IVD hebt die Bedeutung von in medizinischen Laboratorien eigenentwickelten Untersuchungsverfahren für die Patientenversorgung vor allem im Bereich seltener Erkrankungen und neuer diagnostischer Marker hervor. Die IVDR bildet für die Entwicklung und Verwendung von IVD aus Eigenherstellung einen passenden regulatorischen Rahmen, sofern die Anforderungen zuverlässig entsprechend dem Stand der medizinischen Wissenschaft und Technik, aber zugleich pragmatisch und in Übereinstimmung mit den in den Mitgliedstaaten bewährten Qualitätsmanagementsystemen umgesetzt werden. In Deutschland sind hier einerseits die verpflichtenden Anforderungen der RiLiBÄK zu nennen. Andererseits können Elemente von freiwillig anzuwendenden internationalen Normen dazu dienen, die nach Anhang I der IVDR umzusetzenden Anforderungen an Sicherheit und Leistung für IVD aus Eigenherstellung zu erfüllen. Sowohl die Komplexität als auch Lösungskonzepte zur Umsetzung der Anforderungen werden u.a. am Beispiel der erforderlichen Dokumentation, der Leistungsbewertung und der ggf. durchzuführenden Softwarevalidierung aufgezeigt.

Die Ad-hoc-Kommission empfiehlt, bei einer möglichst weitreichend harmonisierten Interpretation der Anforderungen gleichzeitig die für die Patientenversorgung notwendige Flexibilität in der labordiagnostischen Versorgung einschließlich der Verwendung von IVD aus Eigenherstellung zu gewährleisten.

## Introduction

The Association of the Scientific Medical Societies in Germany (AWMF), an umbrella organization of 179 medical societies, which, in turn, represent around 280,000 members, regards the harmonization of the legal framework for conformity assessment and placing in-vitro diagnostic medical devices (IVD) on the market in Europe as fundamentally positive. Regulation (EU) 2017/746 (IVDR) [1], which will fully apply from May 26, 2022, aims to ensure a smoothly functioning internal market as well as a high level of quality for IVD through appropriate standardized measures in the interest of patient safety.

IVD are essential for the early detection, diagnosis, prognosis, and monitoring of diseases, especially communicable, rare, and/or genetic diseases. They also provide information on physiological/pathological conditions and are increasingly being used for the allocation of treatment as part of precision medicine. Due to the lack of commercially available diagnostic medical devices, medical laboratories in a wide range of fields rely almost exclusively on self-developed test methods – these are also called “in-house tests” or “laboratory developed tests” (LDT) –, particularly for diagnosing rare diseases (“orphan diseases”).

From a medical standpoint, assuring patient care is just as important as regulations on quality assurance and product safety. It can be assumed that the commercial marketing of niche products, such as IVD for diagnosing orphan diseases, is not profitable due to the significantly higher costs for approval imposed on manufacturers by the IVDR requirements coupled with the low market revenues that they can expect. For this very reason, health institutions, such as medical laboratories, are compelled to continue to rely on their own in-house manufactured IVD to prevent an undersupply of patient care and to flexibly respond to the special needs or unexpected situations that arise in individual and public healthcare.

## The new IVDR

The previous EC Directive 98/79/EC (IVD Directive, IVDD) did not stipulate any requirements for products manufactured in health institutions for use in that same environment [2]. Due to the EU principle of subsidiarity, requirements for IVD manufactured in-house have been anchored at the national level in the Ordinance on Medical Devices (Section 5 (6) MPV) in conjunction with Section 12 of the Medical Devices Act (MPG) since 2007 [3], [4]. The operation and use of all examination procedures are regulated through national law in Germany by the Medical Devices Operator Ordinance (MPBetreibV) [5] and the MPV. The Guideline of the German Medical Association on Quality Assurance in Medical Laboratory Examinations (RiliBÄK) regulates the basic requirements for the structural and process quality of medical laboratory examinations in Germany [6].

**Figure 1:**
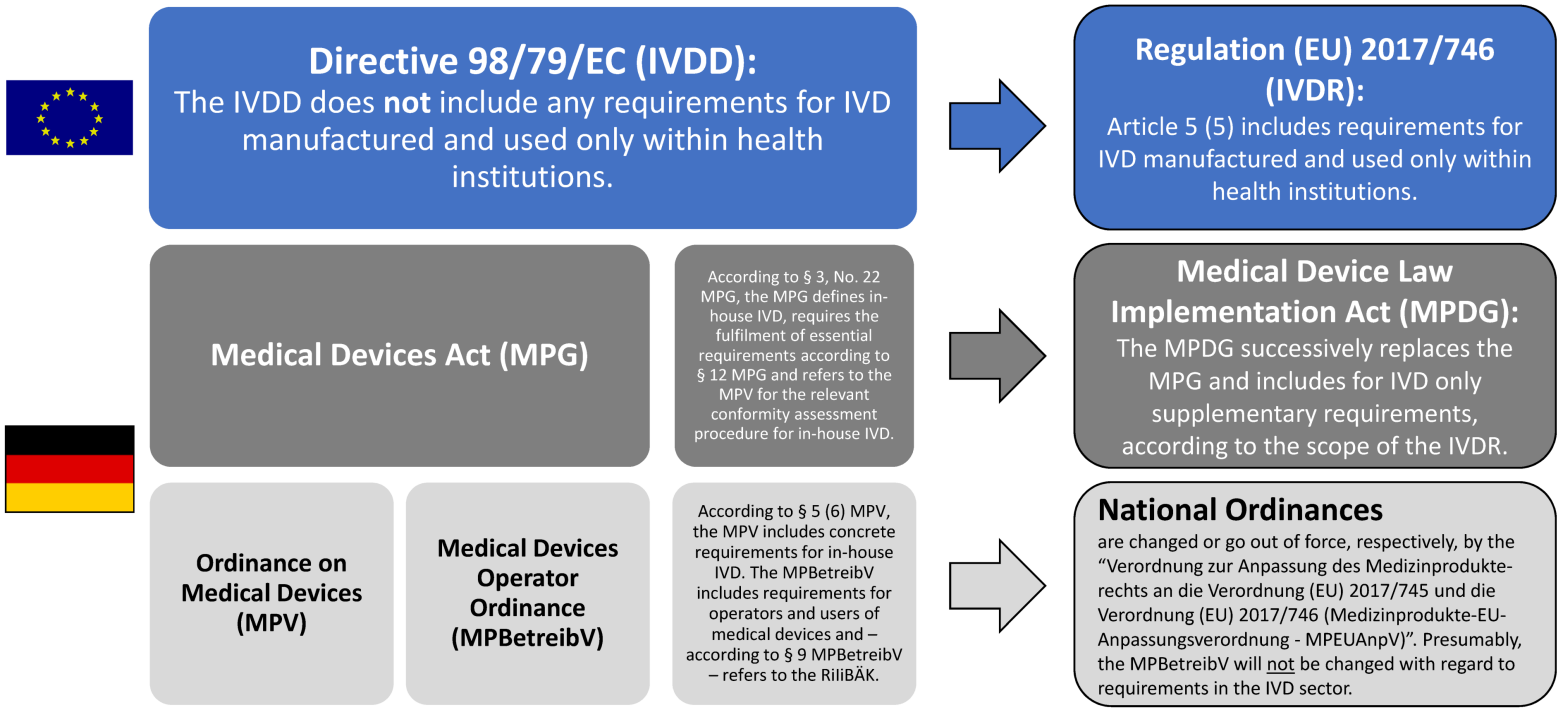
The European and national legal framework for IVD manufactured in-house. The IVDD will be completely replaced by the IVDR on May 26, 2022. In contrast to the IVDD, the IVDR is directly applicable to all addressees and contains requirements for IVD manufactured in-house. The Medical Devices Act (MPG) will gradually be replaced by the Medical Device Law Implementation Act (MPDG), which will only contain regulations that are within the scope of the national legislator. Based on current knowledge, the Medical Devices Operator Ordinance (MPBetreibV) will not be amended for the IVD sector, which will continue to refer to the RiliBÄK. The list of national regulations in the figure is not exhaustive.

The IVDR now harmonizes for the first time throughout Europe the requirements for the manufacture and use of IVD manufactured in-house (Figure 1 (Fig. 1)). The IVDR, however, does *not* claim to regulate diagnostic medical procedures. This is in line with Article 168 of the EU Treaty, according to which the activities of the European Union shall complement the responsibilities of the member states with respect to the organization and delivery of health services and medical care.

According to the new EU Regulation, health institutions located in the EU may continue to manufacture and use self-developed diagnostic products, provided they meet the provisions of Art. 5 (5) of the EU Regulation. However, the scope of some of the requirements has increased over those stipulated in the MPV so that medical laboratories are faced with considerably more effort in terms of validation and documentation and, at times, in implementing standards from areas previously outside their discipline. Furthermore, the EU Commission has so far missed the opportunity to specify the requirements for health institutions that use IVD manufactured in-house, and to clearly distinguish these requirements from those for economic actors, distributors, manufacturers and importers.

## Recommendations of the AWMF Ad hoc Commission In-vitro Diagnostic Medical Devices (IVD)

The AWMF Ad hoc Commission In-vitro Diagnostic Medical Devices (IVD) advocates for an approach that is not only cautious and reliable but also pragmatic and measured when implementing the key aspects of the IVDR requirements in medical laboratories. The requirements and conditions of Art. 5 (5) of the IVDR, which health institutions must fulfill if they manufacture and use IVD products themselves, are explained below. The complexities and the regulatory flexibility are also indicated alongside pragmatic approaches to implementing the requirements. 

### Stipulation of Art. 5 (5), final sentence: This paragraph shall not apply to products that are manufactured on an industrial scale

IVD manufactured in-house should only be exempt from requiring a CE marking if they are not manufactured on an “industrial scale”.

This requirement and/or restriction is identical in content to the currently valid definition for in-house manufactured IVD in Section 3 (22) of the German Medical Devices Act (MPG).

Both the IVDR as well as the MPG, which is still currently in force in Germany, lack a definition or interpretation of the term “industrial scale”. It can be assumed that the intention of such a provision is that IVD that are manufactured in-house may not be produced on a commercial scale using the technical production equipment and facilities required for this. This is because it would imply that they could be directly compared with the production of IVD that are commercially marketed on the basis of conformity assessment procedures and which received a subsequent CE marking.

A similar distinction is made in pharmaceutical law (Section 4 (1) of the Medicinal Products Act AMG) – namely through the use of an “industrial process” – between so-called finished medicinal products, which require marketing authorization, and prescription-based medicinal products manufactured in pharmacies, which usually do not require marketing authorization [7]. Medicinal products that are not manufactured in advance are only considered to be finished medicinal products subject to authorization if an “industrial process” is used to prepare them. However, according to such a legal interpretation, this requires:

1. broad-based manufacturing on a large scale
2. in line with standardized specifications which
3. requires industrial-scale production facilities and equipment,

which is seldom the case in pharmacies [8], [9].

**Conclusion:** The AWMF Ad hoc Commission IVD recommends that the term “industrial scale” be interpreted uniformly at the European level (Medical Device Coordination Group, MDCG) and the national level (Working Group on Medicinal Products AGMP or the corresponding expert group of the Central Authority of the Länder for Health Protection, ZLG) so as to ensure individualized and flexible patient care by medical laboratories that use IVD manufactured in-house.

In this context, the regulations based in pharmaceutical law can serve as a suitable foundation for defining “industrial scale” manufacturing as being:

1. broad-based manufacturing on a large scale,
2. in line with uniform specifications with regard to the manufacturing process, including packaging, labeling and batch documentation,
3. using industrial production equipment and facilities,

which is seldom the case in medical laboratories.

The AWMF Ad hoc Commission IVD is of the opinion that the industrial-scale use of IVD manufactured in-house does not generally apply to medical laboratories.

### Stipulation of Art. 5 (5) (a): The products are not transferred to another legal entity

According to the current understanding and interpretation of this IVDR requirement, test methods that are developed, validated and manufactured in the company’s own laboratory may not be transferred to another legally independent facility, in other words, to another legal entity.

Medical laboratories in Germany have various organizational and legal forms. They are organized as individual laboratories, group practices, laboratory communities or in a community health center with the legal forms GbR, PartG, GmbH, or as a laboratory association, or they are integrated into larger hospital networks with correspondingly diverse legal ownerships. Medical laboratories operate as independent entities in communities, partnerships and alliances.

Ensuring good medical care requires structures that are in line with a physician’s perception of practicing medicine. For this reason, new legal forms and forms of medical practice have been introduced in Germany, also with the aim of bundling synergies to achieve high-quality, quality-assured and cost-efficient patient care. This intention could be counteracted by a possible interpretation of the IVDR stipulation in which medical laboratories that are contractually bound within an organizational structure and legal form, but which operate independently, can neither use an IVD product developed and manufactured in that environment, nor benefit from a reduction in the administrative and documentation burden involved with implementing the IVDR requirements.

**Conclusion:** The AWMF Ad hoc Commission IVD recommends that medical laboratories within an organizational and legal form be regarded as a unit and therefore be allowed to exchange their products with each other so long as the requirement that the products remain within one legal entity is met and the IVD is not commercially marketed or otherwise placed on the market.

This does not affect a laboratory’s duty to demonstrate the successful transfer of a testing procedure as part of a technology transfer.

### Stipulation of Art. 5 (5) (b): A quality management system appropriate for the manufacture and use of the devices

Within the scope of this requirement, the IVDR does not define the form of quality management system that must be established by the health institution or, where applicable, the subordinate organizational unit of the health institution, such as the medical laboratory located within it.

In either case, legal requirements currently exist throughout Europe with respect to the implementation of a QM system in virtually all forms of health institutions. These have been implemented in Germany for years, for example, through the provisions of Section 135a of the German Social Code (Sozialgesetzbuch V). However, it is debatable whether these QM systems are always suitable for the production and use of IVD manufactured in-house.

However, further elaboration on the characteristics of the QM system of health institutions is not considered necessary since IVD that are manufactured in-house are nearly always produced and used by medical laboratories and their requirements are formulated further on in Article 5 (5) (c) of the IVDR.

**Conclusion:** The AWMF Ad hoc Commission IVD recommends that the existing European regulations and the national regulations of the individual member states stipulating the requirements for QM systems of health institutions be recognized and, if necessary, further developed. Coexistence or integration of the medical laboratory’s QM system into that of the health institution is considered to be principally feasible based on previous experience.

### Stipulation of Art. 5 (5) (c): The laboratory of the health institution is compliant with Standard EN ISO 15189 or, where applicable, national provisions including national accreditation provisions

The AWMF Ad hoc Commission IVD is of the opinion that the Article 5 (5) (c) of the IVDR does not clearly outline the quality requirements and the competencies of medical laboratories that manufacture and use devices within a health institution and that there is a large degree of ambiguity. On the one hand, laboratories are required to conform to the standard EN ISO 15189 [10], on the other hand the term “or” places this on an equal footing with national regulations, including national accreditation regulations.

EU member states have heterogenous requirements for QM systems and medical laboratory accreditation. This ranges from a legally required “full accreditation” in accordance with EN ISO 15189 to voluntary accreditation programs. In Germany, all medical laboratories have been required to implement a QM system since the creation of the RiliBÄK and its anchoring in the Medical Devices Operator Ordinance (MPBetreibV) in 2008. At the same time, more than 400 medical laboratories are accredited according to EN ISO 15189 (and comparable standards such as EN ISO 17020), mostly on a voluntary basis; accreditation is mandatory for only a few of these laboratories (for example, in the area of newborn screening).

The requirements of EN ISO 15189 with regard to validation, verification and quality assurance of examination procedures, which also include “non-standardized procedures” and “procedures designed or developed for the laboratory”, correspond to the state of the art in medical science and technology and ensure that an international level of quality is achieved in this field. In addition, the requirements for the examination procedures, etc., are flanked by elements of a QM system recognized by EN ISO 15189, such as documentation requirements, and requirements for implementing corrective and preventive actions.

Many of the requirements of EN ISO 15189 are reflected in the RiliBÄK, but there are also differences. In terms of the quality of results, the RiliBÄK formulates some criteria that are not found in EN ISO 15189. No reference to EN ISO 15189 is made in the RiliBÄK.

**Conclusion:** The AWMF Ad hoc Commission IVD recommends that laboratories implement the concepts for QM systems stipulated and established by the respective member states for all areas of activity affecting IVD manufactured in-house. Implementation can be demonstrated, for example, through voluntary accreditation of the laboratory in accordance with international standards or by compliance with national regulations such as the RiliBÄK. Independently of this, the QM system is inspected by the competent authority responsible for surveillance (in Germany: state authorities such as the trade supervisory office, state office for social services, bureau of standards, etc.), so that conformity with requirements can also be demonstrated within the framework of this surveillance.

### Stipulation of Art. 5 (5) (d): Justification that a target patient group’s specific needs cannot be met or cannot be met at the appropriate level of performance by an equivalent device available on the market

Neither the IVDR nor the previously published European or national regulatory guidance on the IVDR contain explanations of the term “equivalence”. The IVDR uses the terms “equivalent” and “similar” in several passages without making a clear distinction. Currently there is only a statement by the UK authority MHRA that can be used to interpret the “justification” requirement [11]; guidance issued by MedTech Europe, the European trade association for the medical technology industry, interprets the equivalence principle in terms of the principles of performance evaluation for commercially available in-vitro diagnostic medical devices [12].

However, the requirement under Article 5 (5) (d) of the IVDR refers to the “indicated level of performance” or the “specific needs of the target patient group”. Accordingly, a variety of analytical and clinical-diagnostic performance characteristics of the IVD in question are considered in the context of this comparison, which are also determined by the method, design, available controls, turn-around times, experience from long-term use, etc. However, many of these characteristics are not published for commercially available, CE-marked IVD and, therefore, medical laboratories using IVD that are manufactured in-house can neither access nor assess these characteristics by means of the available literature.

Furthermore, medical laboratories must be responsive to the requirements of attending physicians and be specific (rather than “similar”) in what they offer in order to meet the demands of complex patient care.

**Conclusion:** The AWMF Ad hoc Commission IVD recommends that a harmonized understanding of the “equivalence” of IVD and the possible characteristics that should be considered be achieved and communicated on a European (MDCG) or national level (Working Group on Medicinal Products, AGMP, or the corresponding expert group of the Central Authority of the Länder for Health Protection, ZLG).

It is also recommended that, in the interest of ensuring patient-centered care, the medical laboratories using IVD manufactured in-house should be solely responsible for selecting *the applicable characteristics in the respective cases* when assessing equivalence, to document these clearly and plausibly, and to submit them to the competent surveillance authority upon request.

### Stipulation of Art. 5 (5) (e): Providing information about the device to the competent authority including justification for its manufacturing, modification and use

The IVDR requires that medical laboratories prepare documentation that provides an understanding of the manufacturing site and process, design, and performance data of the device, including its intended use, and to provide this to their competent surveillance authority upon request. This information largely corresponds to the contents of the technical documentation for demonstrating compliance with the general safety and performance requirements of the IVDR.

The IVDR has a separate annex on technical documentation (Annex II), which specifies in a clear, organized, readily searchable and unambiguous manner the extensive elements needed to describe the device’s intended purpose and how to ensure that this performance is actually being provided. Health institutions that manufacture and use IVD products themselves are not required to prepare technical documentation to the full extent as outlined in Annex II. Here the IVDR does not explicitly specify any requirements.

**Conclusion:** In order to create – despite the federal structure – the clearest and most uniform specifications possible in Germany with regard to the scope and format of the documentation, the AWMF Ad hoc Commission IVD seeks to develop a generally applicable, streamlined and expedient “document format” which health institutions that use in-house devices can use to provide the required information. This should meet the requirements of the IVDR, however it should not present health institutions with the challenge of not being able to perform, or only to a limited degree, their actual task of providing healthcare to patients due to massively increased documentation requirements.

### Stipulation of Art. 5 (5) (i): Review of the experience gained from clinical use of the devices, taking all necessary corrective actions

Commercial IVD manufacturers are required by EC Directive 98/79/EC – and health institutions with in-house manufacturing are required by the National Ordinance on Medical Devices – to establish a systematic process for evaluating experience with the use of their IVD devices and to initiate any necessary corrective measures. Neither the EU Directive nor the German ordinance has specified the design of such a post-market surveillance system (PMS). The manufacturer/“in-house manufacturer” has been tasked with implementing a concept adapted for the IVD. 

In the recitals to the IVDR, the EU Commission now states that a set of instruments for monitoring the clinical efficacy and safety of the IVD in use on the market must be established with and through the QM system. This is regulated in detail for commercial manufacturers in Chapter VII Section I and Annex III of the IVDR. The PMS strategy should be a component of the QM system. EN ISO 13485 and EN ISO 14971 already require this of medical device manufacturers [13], [14].

**Conclusion:** The IVDR does not expressly require such a PMS process for health institutions that use products developed and manufactured in-house for diagnostic purposes. What is required is a documented assessment of experience with the products during routine in-house use and any necessary corrective measures to be derived from this.

These processes are already an integral part of EN ISO 15189, which is sufficient for medical laboratories. They are referenced in the standard in many places, particularly with regard to a regular management assessment of the suitability and clinical effectiveness of examinations as well as patient safety, and with respect to corrective and preventive measures and external evaluations, both in terms of accreditation and as part of participation in a performance evaluation review.

The AWMF Ad hoc Commission IVD aims to provide guidance for medical laboratories on the regulations of the RiliBÄK and EN ISO 15189 already in existence, which shows which methods are used for the evaluation of data obtained in routine diagnostic testing, which specifications can be defined as the basis for deciding if and which CAPA measures are be initiated, and how these processes and results can be documented for the surveillance authorities.

### Stipulation of Art. 5 (5): Fulfillment of the relevant general safety and performance requirements set out in Annex I

#### Annex I Chapter 1: Risk management

Annex I, Section 3 of the IVDR requires the implementation, documentation and maintenance of a risk management system. Risk management is understood in this context as being a “continuous, iterative process throughout the life cycle of a device” that requires “regular systematic updating”.

Commercial manufacturers usually apply the harmonized standard EN ISO 14971:2013 in the context of risk management; this standard was recently revised and published – without harmonization with the IVDR – as EN ISO 14971:2020. Corresponding to its area of application, EN ISO 14971 uses terminology that is rarely applied in a medical laboratory context.

However, EN ISO 22367 was published in September 2020 which specifically addresses risk management in medical laboratories. It defines a risk management process that medical laboratories can use to identify and manage risks associated with medical examinations which affect patients, laboratory staff and service providers [15]. The process includes the identification, estimation, assessment, control and monitoring of risks as required by the IVDR, and also takes into account aspects of the pre- and post-analytical phases.

**Conclusion:** The AWMF Ad hoc Commission IVD recommends that Standard EN ISO 14971 should not apply to medical laboratories.

Instead, the criteria in EN ISO 22367:2020, which are applicable to examination procedures that medical laboratories develop for their own use, may provide a suitable basis for the risk management required under the IVDR.

#### Annex I Chapter II (9): Performance characteristics

According to the definition of the IVDR (Article 2 (39)), the performance of a device refers to its ability to achieve the intended purpose as claimed by the manufacturer. This consists of the analytical performance and, where applicable, the clinical performance supporting its intended purpose. The performance characteristics for both the design and manufacture of a device are listed in Annex I. There are no further requirements with respect to the scope and verification of performance characteristics.

However, the IVDR expands the requirements for demonstrating performance, as Article 5 of the IVDR states that demonstration of conformity with the general safety and performance requirements set out in Annex I shall include a performance evaluation. In turn, a performance evaluation (clinical evidence) is defined as an assessment and analysis of data to establish or verify the scientific validity, the analytical performance and, where appropriate, the clinical performance of a device (Article 2 (44) IVDR). Another term is introduced here, ‘scientific validity’, which illustrates the association of analyte and clinical picture or physiological state. The performance evaluation then provides clinical evidence that a product is safe and achieves the intended clinical benefit.

Focus is on clinical performance and scientific validity in addition to validating the analytical procedure, which includes the experimental design, plan and assessment report. Clinical performance demonstrates the diagnostic accuracy of the IVD and depends on the intended purpose and thus on a defined patient population. The IVDR requires extensive performance studies to assess clinical performance characteristics as part of the clinical evidence. Scientific validity, which demonstrates the association of an analyte with a clinical condition or physiological state, requires a systematic literature search. This may include literature data, expert opinions, position statements, and guidelines from professional societies. The documentation should contain a comprehensible search strategy and algorithms, sources used, and criteria on selecting data. 

**Conclusion:** For medical laboratories, the requirements of the RiliBÄK and EN ISO 15189 apply with respect to demonstrating performance. They contain all the criteria specified in Annex I, Chapter 2 of the IVDR for a comprehensive validation of diagnostic test kits. An exception is made for the clinical performance studies required for evaluating the clinical performance characteristics. Here, the EU regulation provides latitude to refrain from conducting clinical performance studies if there are enough reasons to rely on other sources of clinical performance data. This can include scientific literature as well as data collected from published routine diagnostic tests that have been conducted in an in-house laboratory.

The AWMF Ad hoc Commission IVD recommends that health institutions first specify and substantiate the scope of the clinical evidence of their devices in the documentation integrated into their QM system in order to demonstrate compliance with the general safety and performance requirements. For the performance evaluation of the IVD product, results on the scientific, analytical and clinical performance are then summarized in a report, demonstrating the positive risk-benefit ratio.

#### Annex I Chapter II (16): Software

IVD software includes software that is an independent IVD (stand-alone software), a part of an IVD (embedded software) or an IVD accessory. This includes software solutions for evaluating the measurement results of testing procedures, applications for calculating and interpreting findings, and laboratory information systems, which usually have to be adapted to the laboratory processes and other IT infrastructure (interfaces) in order to be used in the medical laboratory. Combinations of software are quite common here in order to map the overall process. For example, an analysis device can communicate as an IVD with a middleware or a laboratory information system, which can send the data (modified if necessary) back to a hospital information system (potentially a medical device).

Especially in terms of state-of-the-art high-throughput methods, where the speed of technical development as well as scientific and clinical findings is immensely high (e.g. next generation sequencing (NGS) technology), medical laboratories depend on parameterization, configuration, customization and on completely self-developed software tools, where the boundaries to commercial software (IVD or medical device) are fluid. In addition, there are hybrid forms, especially in bioinformatics pipelines (calling, annotation, variant assessment), in which software components from commercial manufacturers are adopted and external data sources are used alongside data from service providers with self-developed systems and freely accessible (open source) software.

The IVDR and MDCG Guidance 2019-11 [16] do not address the issue of software manufactured and used in a health institution and leave many questions unanswered regarding the distinctions between that which is developed in-house and that which is placed on the market. Furthermore, some aspects, such as the increasingly important field of bioinformatics, are not being taken into account.

The aforementioned requirements apply similarly to software, including the stipulations that no comparable software product exists on the market, that the general safety and performance requirements of Annex I must be met, and that technical documentation must be generated. According to Annex I Chapter 2 (16), the software development must take into account the software lifecycle process and risk management, which includes information security, verification and validation. Furthermore, minimum requirements need to be specified concerning hardware, IT network characteristics and IT security measures including protection against unauthorized access. The required documentation should set out the risk classification and the development stages of the software, describe the data processing and data evaluation, particularly of the algorithms used, and summarize the verification and validation, taking into account the usage environment, hardware configurations and possible operating systems.

**Conclusion:** The AWMF Ad hoc Commission IVD recommends revising the existing MDCG Guidance on qualification and classification of software by integrating software used for diagnostic testing that has yet to be considered, such as bioinformatics pipelines or combinations of software. Furthermore, a clarification is required specifically for software developed in-house by the health institutions which:

- differentiates between the stringent performance evaluation requirements for software or pipelines of commercial manufacturers, and software not marketed by health institutions,
- unambiguously defines the boundaries between self-developed, parameterized and configured software,
- pays attention to the specific needs of medical laboratories when integrating software developed in-house or software modules of commercial systems.

## Abbreviations

- AGMP: Working Group on Medicinal Products of the ZLG
- AMG: Medicinal Products Act
- AWMF: Association of the Scientific Medical Societies in Germany
- BAG: Group practice
- FEG: Expert group of the ZLG
- GbR: Partnership under civil law
- GmbH: Limited liability company
- IVD: In-vitro diagnostic medical devices
- IVDD: EU Directive on In-vitro Diagnostic Medical Devices (98/79/EC)
- IVDR: European In-vitro Diagnostic Medical Device Regulation ((EU) 2017/746)
- LDT: Laboratory Developed Test
- MDCG: Medical Device Coordination Group
- MHRA: Medicines and Healthcare Products Regulatory Agency
- MPBetreibV: Medical Devices Operator Ordinance
- MPG: Medical Devices Act
- MPV: Ordinance on Medical Devices
- MVZ: Community health center
- NGS: Next Generation Sequencing
- PartG: Partnership company
- PMS: Post-market surveillance
- RiliBÄK: Guideline of the German Medical Association on Quality Assurance in Medical Laboratory Examinations
- ZLG: Central Authority of the Länder for Health Protection with regard to Medicinal Products and Medical Devices

## German version

The German version of this position paper was published in GMS Zeitschrift zur Förderung der Qualitätssicherung in medizinischen Laboratorien [17].

## Competing interests

The authors declare that they have no competing interests.
